# Zero Echo Time MRI for Osseous Assessment of Sports-Related Pathology in Athletes: A Pictorial Essay

**DOI:** 10.5334/jbsr.4198

**Published:** 2026-02-16

**Authors:** David F. Hanff, Filip M. Vanhoenacker, Edwin H.G. Oei

**Affiliations:** 1 Department of Radiology and Nuclear Medicine, Erasmus Medical Center, Rotterdam, The Netherlands; 2 Department of Radiology, AZ Sint-Maarten, Mechelen, Belgium; 3 University Hospital Antwerp, Faculty of Medine and Health Sciences, Universities Antwerp and Ghent, Belgium; 4 Department of Radiology and Nuclear Medicine, Erasmus Medical Center, Rotterdam, The Netherlands

**Keywords:** sports imaging, zero echo time (ZTE), CT-like bone imaging, athletic injuries, advanced imaging

## Abstract

Assessment of cortical bone and mineralized tissue in athletes traditionally relied on radiographs and CT, as conventional MRI provides limited/no signal from cortical bone. Recent advances, particularly zero echo time (ZTE) MRI, enable CT-like visualization of osseous structures within routine MRI protocols. This pictorial essay highlights the added value of ZTE in sports imaging, illustrating its role in the evaluation of stress injuries, occult fractures, osteoid osteoma, osteochondral lesions, calcified tendon pathology, hip and groin pathologies, and relevant differential diagnosis. ZTE complements conventional MRI by improving the depiction of cortical and mineralized abnormalities relevant to athletic populations.

## Introduction

Bone or mineralized tissue imaging in musculoskeletal radiology traditionally relied on radiographs and computed tomography (CT) to depict cortical structures, fractures, and osseous morphology. Radiographs provide fast, low-cost assessment but are limited by superimposition and reduced sensitivity for subtle lesions. CT offers high spatial resolution and is often considered the reference standard for cortical bone evaluation, preoperative planning, and injury characterization in athletes [1, 2].

Conventional MRI, while excellent for visualizing bone marrow, cartilage, and soft tissues, has long been limited in bone assessment. Cortical bone contains very few mobile protons with extremely short $T_{2}^{*}$ relaxation times, resulting in a signal void on standard sequences [3, 4]. As a result, MRI has traditionally been unable to replace CT for accurate cortical bone visualization [3]. Recent advances, including zero echo time (ZTE) and ultrashort echo time sequences, enable capture of rapidly decaying signals, thereby producing CT-like bone contrast directly on MRI [3, 5, 6]. In addition to short echo-time approaches, mineralized tissue can also be visualized using T1-weighted gradient-echo sequences, which depict bone indirectly through signal suppression of surrounding soft tissues, and susceptibility-weighted imaging, which highlights calcifications based on magnetic susceptibility effects rather than direct signal acquisition [4]. Alternatively, synthetic CT may be obtained using an MRI-only post-processing approach that reconstructs CT-equivalent bone information from conventional MRI data [7].

For athletes, the availability of information regarding the bony structures from a regular MRI examination is particularly compelling. The ability to combine soft-tissue evaluation with osseous detail in a single MRI protocol streamlines workflow and reduces the need for dual-modality imaging. Replacing CT with ZTE in these contexts may also lower costs, simplify scheduling, and accelerate clinical decision-making in both acute and overuse injuries [8].

In this pictorial overview, we illustrate the potential of ZTE MRI to visualize cortical bone and mineralized tissue in athletes, demonstrating how it complements conventional MRI by enabling CT-like assessment of osseous abnormalities within a single examination. We acquired all MR images on 1.5 and 3.0 Tesla scanners (GE HealthCare, Waukesha, Wisconsin, USA).

### Stress injuries or occult fractures

Bone stress injuries or stress fractures are common in athletes, accounting for up to 20% of all sports injuries, particularly in endurance sports with high repetitive loading [9]. Although conventional radiography is commonly used for initial assessment, its sensitivity in early-stage bone stress injuries is low (10%–35%), necessitating MRI for further evaluation [10].

**Figure 1:**
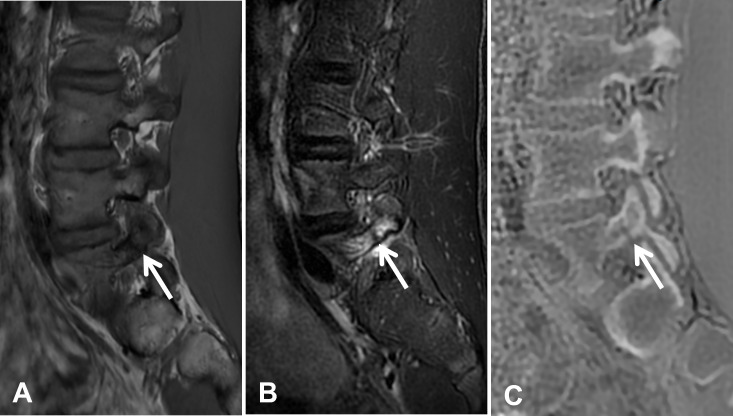
A 15-year-old male with a left L5 pedicle stress fracture. **(A)** Sagittal MR images demonstrate hypointense signal abnormality (arrow) on the T1-weighted image, associated bone marrow edema (arrow) on the fat-suppressed T2-weighted image **(B)** with a linear hypointense fracture line in the left L5 pedicle (white arrow). **(C)** The sagittal ZTE image clearly depicts the cortical discontinuity (arrow), confirming the diagnosis of a stress fracture.

**Figure 2:**
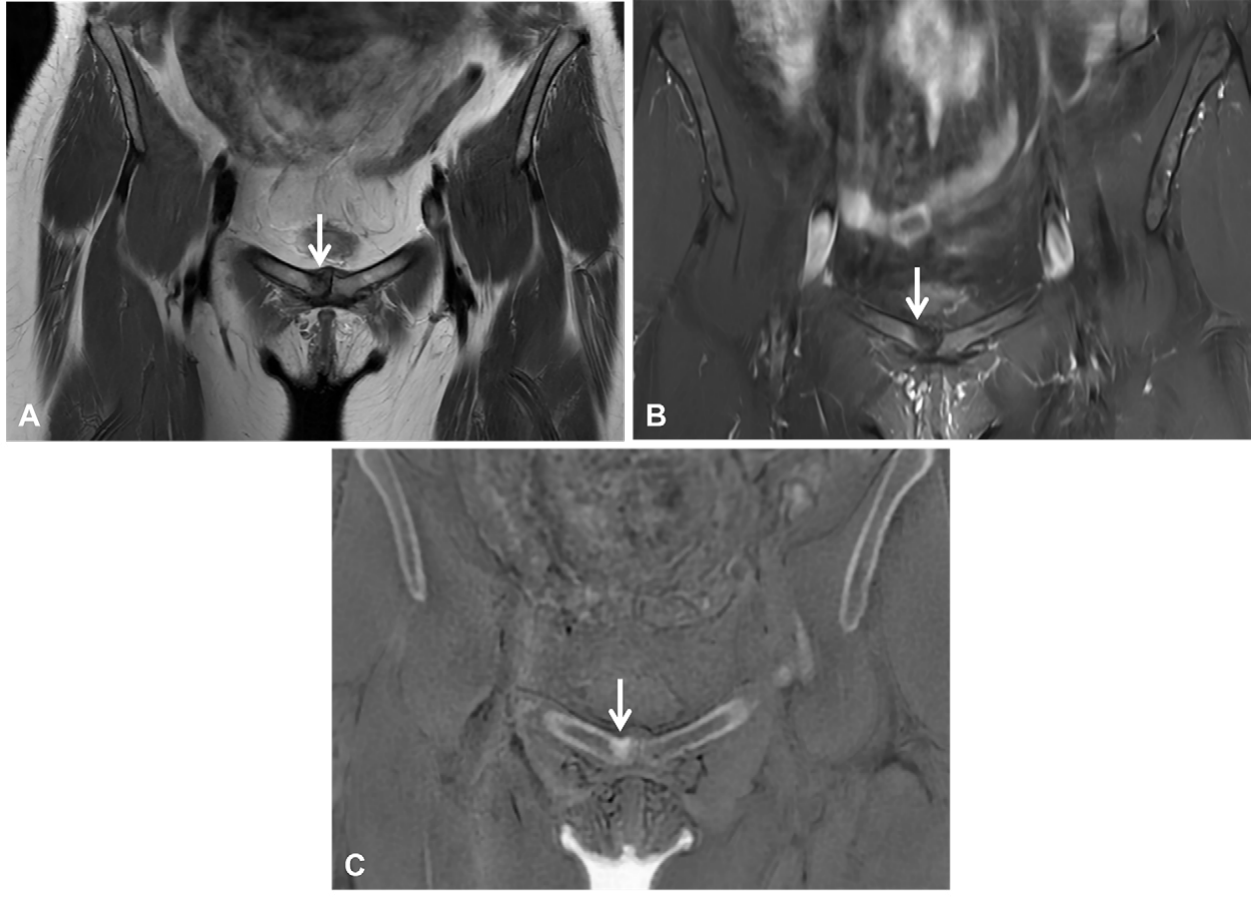
MRI of the pelvis in a 48-year-old female long-distance runner demonstrating a stress injury (arrows). **(A, B)** Coronal T1 and proton-density fat-suppressed images demonstrate signal changes in the pubic symphysis with associated bone marrow edema (arrow). **(C)** The coronal ZTE image demonstrates a sclerotic line compatible with a stress injury.

Figure 1 demonstrates a high-level male padel athlete presenting with lower back pain and a stress fracture of the left pedicle, while Figure 2 illustrates a stress injury in a female long-distance runner.

**Figure 3:**
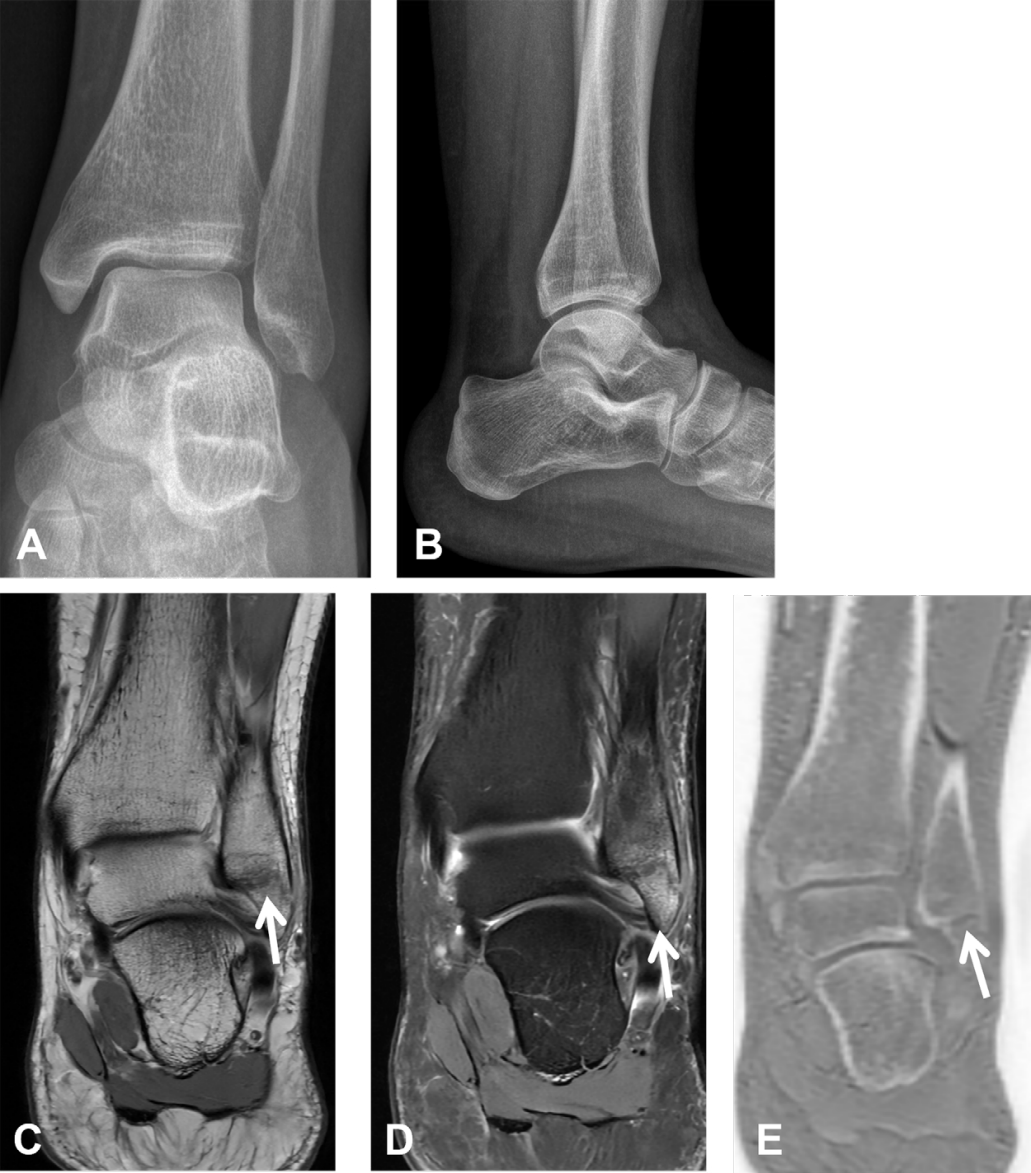
A distal fibular fracture in a 45-year-old female patient following an ankle sprain. **(A, B)** Anteroposterior and lateral ankle radiographs show no definite fracture. **(C, D)** Coronal proton density and coronal fat-suppressed T2-weighted MR images demonstrate a subtle hypointense line in the distal fibula with associated bone marrow edema (white arrows). **(E)** The coronal ZTE image clearly depicts the cortical fracture line.

In addition to stress-related injuries, ZTE imaging may serve as a valuable adjunct in the evaluation of more common traumatic injuries in athletes, such as ankle sprains. As illustrated in Figure 3, a sagittal ZTE image clearly depicts a fibular fracture that was not visible on prior conventional radiographs, and the fracture line was less conspicuous on conventional MRI sequences.

**Figure 4:**
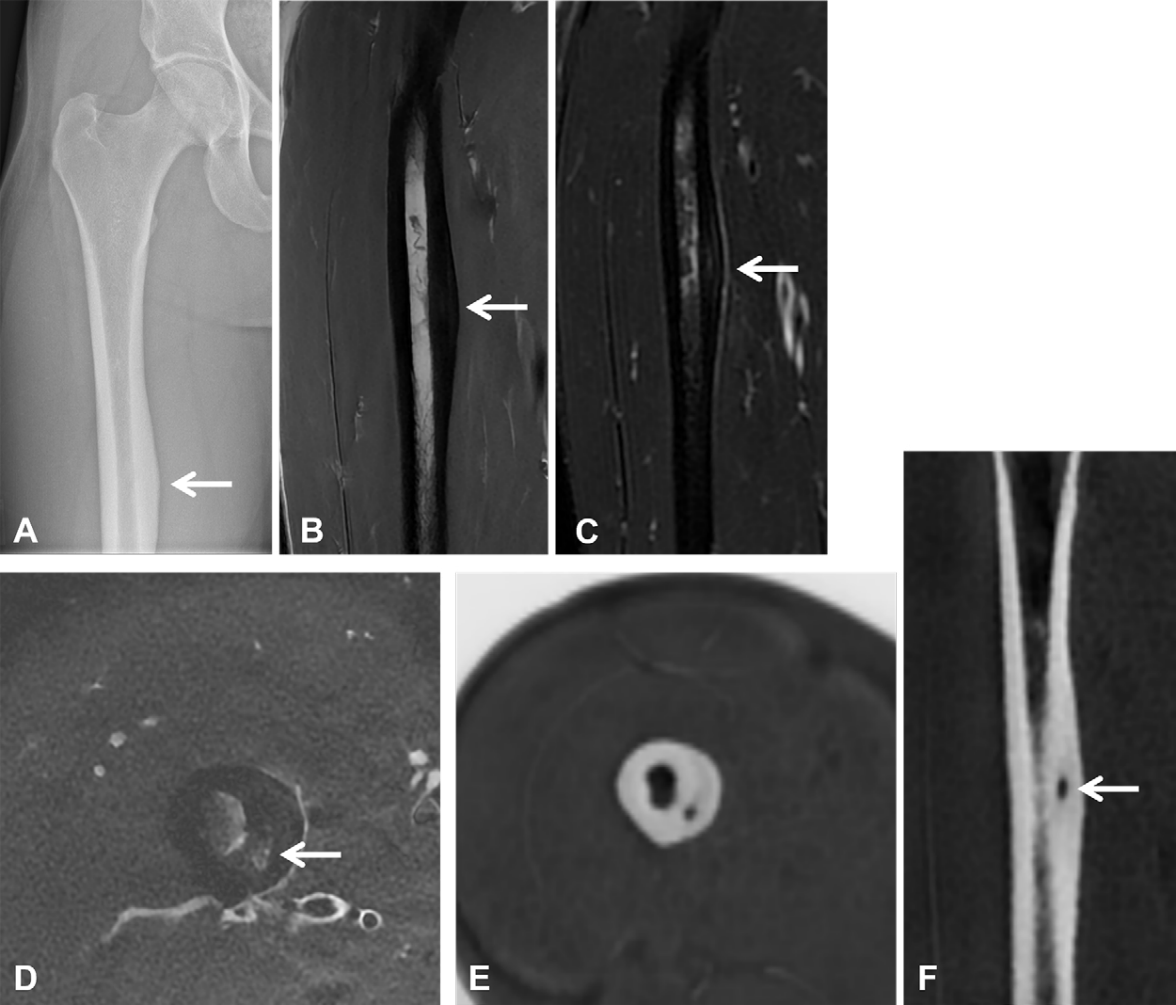
Osteoid osteoma of the right femoral diaphysis in a 23-year-old kickboxing male athlete. **(A)** Anteroposterior radiograph of the right femur demonstrates focal cortical thickening along the medial diaphysis (arrow). **(B, C)** Coronal T1-weighted and fat-suppressed T2-weighted MR images show medial cortical thickening with periosteal reaction (arrows). **(D)** Axial fat-suppressed T2-weighted MR image reveals a subtle central hyperintensity suggestive of a nidus (arrow). **(E, F)** Axial and coronal ZTE images clearly depict a small cortical nidus within the thickened cortex (arrows), confirming the diagnosis of osteoid osteoma.

In younger athletes, clinicians should be aware that osteoid osteoma represents an important differential diagnosis in localized pain. Figure 4 illustrates a 23-year-old male kickboxing athlete presenting with right proximal thigh pain following high-intensity training with a suspected stress fracture. MRI demonstrated cortical thickening with periosteal reaction and a subtle central abnormality suggestive of a nidus. Although CT is typically required to identify the nidus in suspected osteoid osteoma, the ZTE sequence acquired during the same MRI examination clearly depicted the cortical nidus, confirming the diagnosis and obviating the need for additional CT imaging.

### Osteochondral lesions

**Figure 5:**
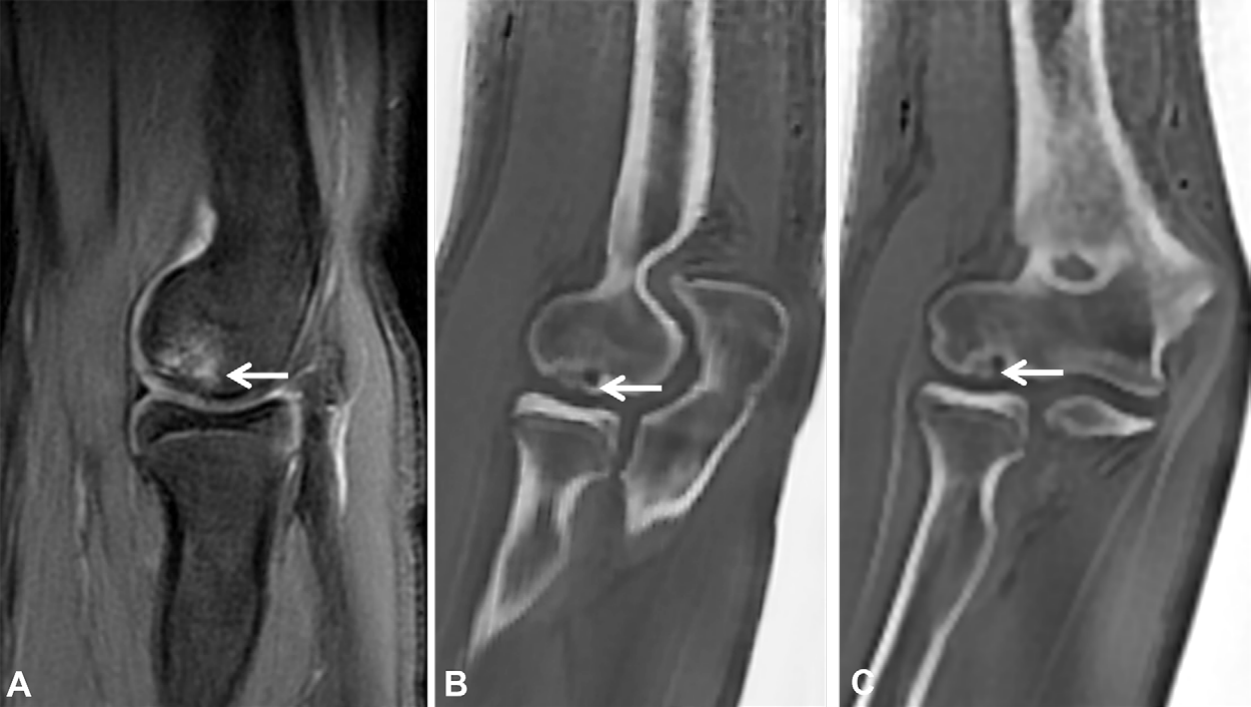
A 15-year-old female gymnast with pain in the elbow. **(A)** Sagittal T2 fat-suppressed MRI demonstrates subchondral bone marrow edema (arrow) in the capitellum accompanied with thinning of the overlying articular cartilage. **(B, C)** Sagittal and coronal ZTE images provide enhanced visualization of the articular surface, depicting contour irregularity and a subtle bony fragment (arrows).

**Figure 6:**
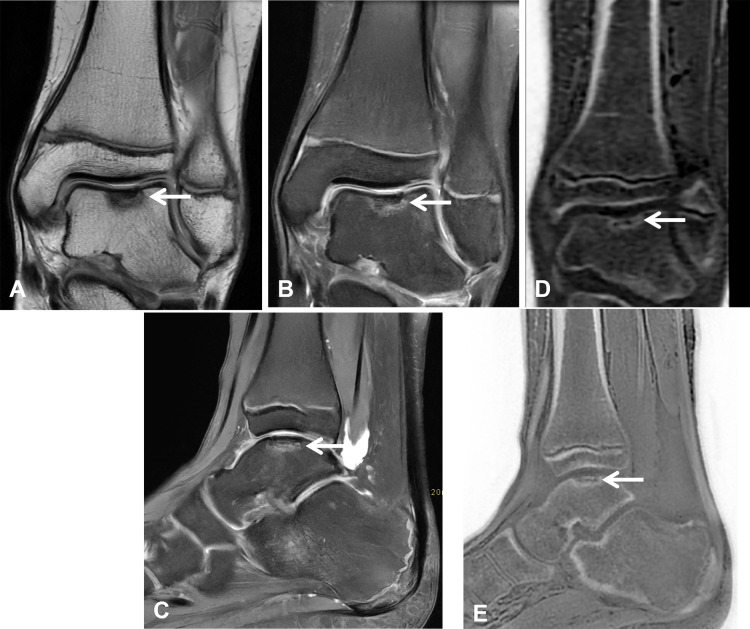
A 12-year-old male soccer player with an ankle injury. **(A)** Coronal proton density without and with fat suppression, **(B)** and **(C)** sagittal proton density fat-suppressed image shows an osteochondral lesion centered in the talar dome (arrow), with associated subchondral bone marrow edema and articular cartilage involvement. **(D, E)** Coronal and sagittal ZTE images demonstrate the centrally cortical fragment with mild loss of height of the subchondral bone (arrow).

**Figure 7:**
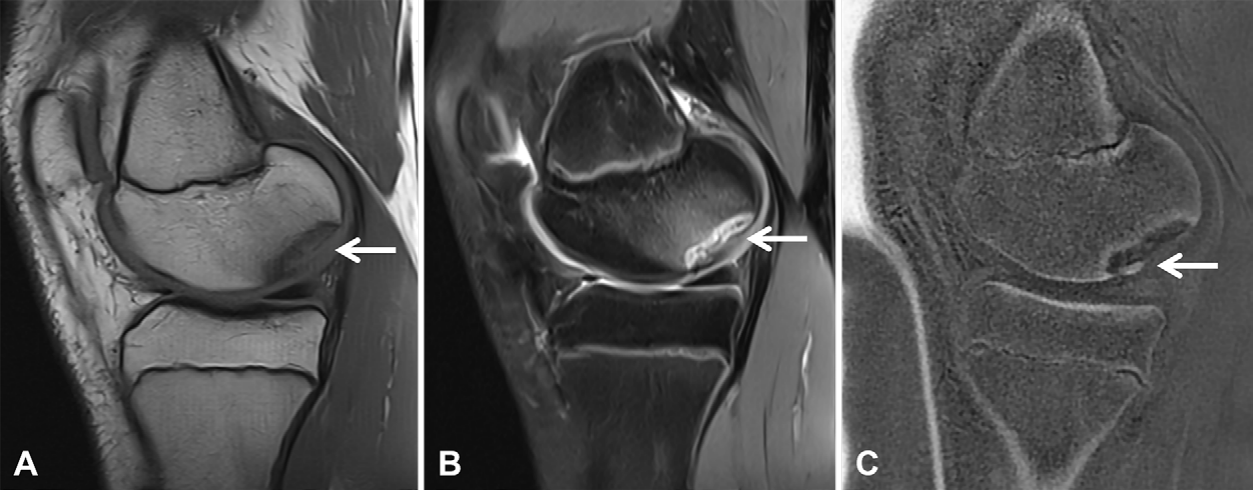
A 15-year-old male soccer player with right knee giving way. Sagittal MRI of the knee demonstrates a large osteochondral lesion (arrows). **(A)** Sagittal T1-weighted and **(B)** fat-suppressed proton density images show a focal osteochondral defect in the medial femoral condyle with associated cartilage involvement and subchondral bone marrow edema (arrows). **(C)** The ZTE image provides improved delineation of the subchondral bone, demonstrating a fragmented cortical fragment (arrow).

Osteochondral lesions, involving injury to the articular cartilage and underlying subchondral bone, are a recognized cause of joint pain and functional limitation in athletes, particularly in young and skeletally immature individuals. They most commonly involve the ankle, the knee, and the elbow, predominantly affecting the capitellum in adolescent overhead athletes [11–13]. Adding the ZTE sequence to the dedicated MRI protocol provides improved visualization of the subchondral bone and cortical architecture, enabling more accurate assessment and staging of osteochondral lesions [14]. Figures 5–7 show three representative examples of osteochondral lesions, involving the elbow in a young gymnast (Figure 5), the ankle in a football player (Figure 6) and the knee in a football player (Figure 7).

### Calcified tendon pathology

**Figure 8:**
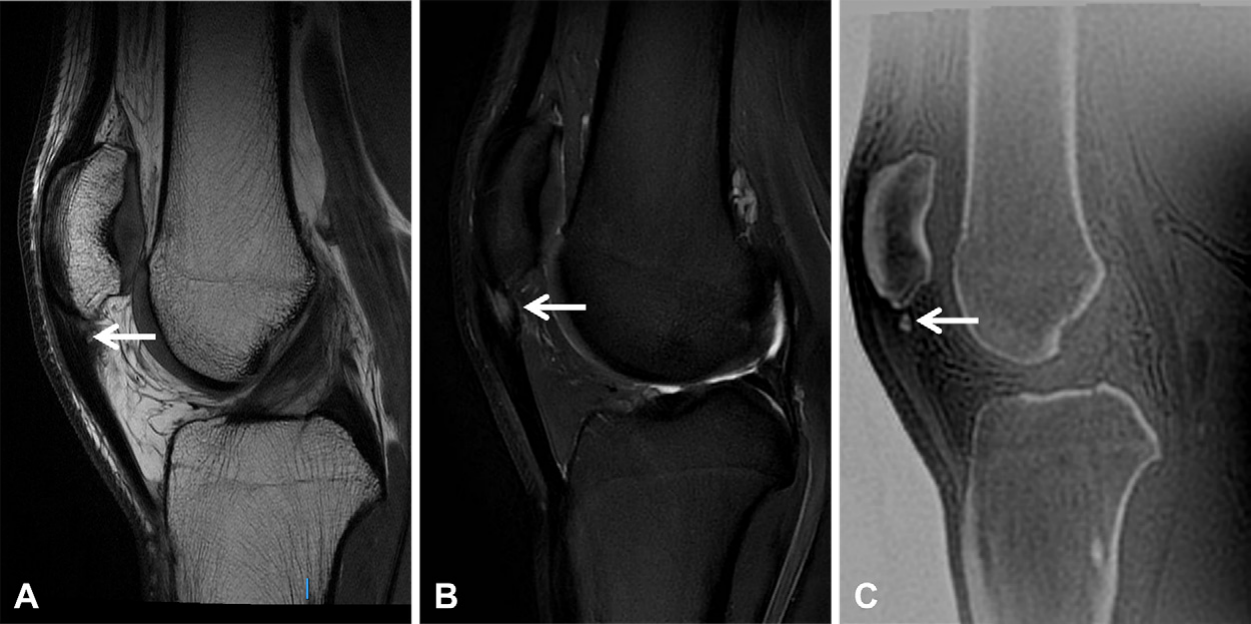
A 24-year-old high-level football player presenting with anterior knee pain, consistent with jumper’s knee. **(A)** Sagittal proton density-weighted and **(B)** T2 fat-suppressed images demonstrate focal thickening of the proximal patellar tendon insertion with increased intratendinous signal intensity (arrows). **(C)** The sagittal ZTE image depicts a small calcification (arrow) that is not appreciable on conventional MRI sequences.

**Figure 9:**
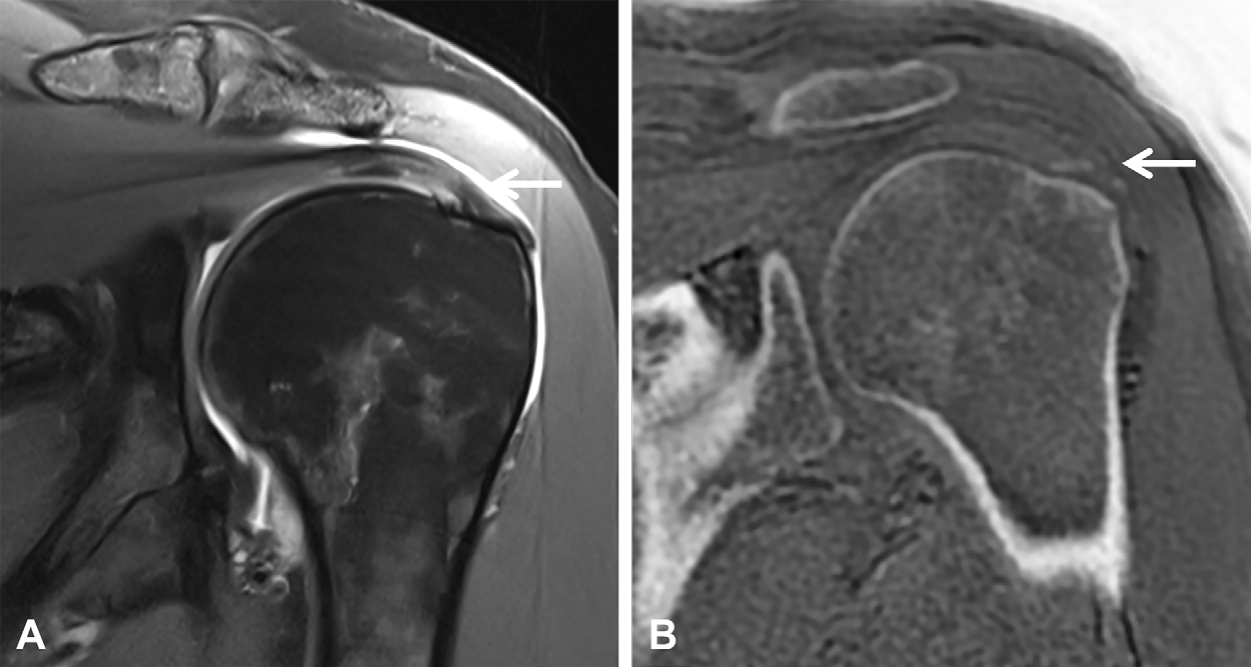
A 52-year-old active female presenting with left shoulder pain and calcific tendinitis. **(A)** The coronal proton density-weighted image demonstrates an inhomogeneous signal at the supraspinatus tendon insertion and increased fluid in the subacromial bursa (arrow). **(B)** The coronal ZTE image clearly depicts the calcific deposits with sharp delineation of the mineralized component (arrow).

Calcified lesions within tendons can be challenging to identify on conventional MRI, as mineralized tissue often appears indistinct or is obscured by surrounding soft-tissue signal. Enhanced depiction of calcification facilitates differentiation from other causes of tendon signal alteration, such as mucoid degeneration or partial tearing (Figures 8 and 9).

### Hip and groin pain

**Figure 10:**
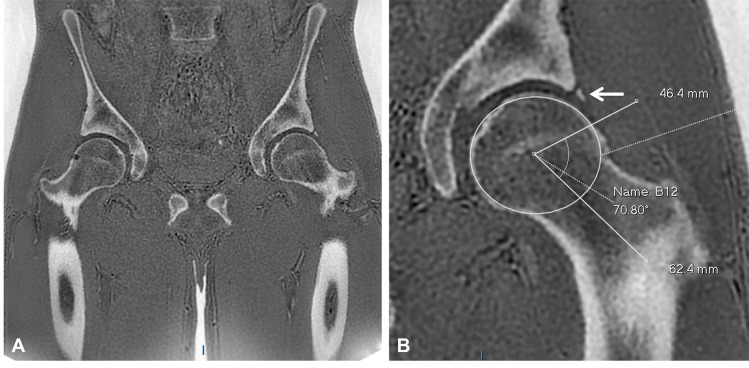
Coronal ZTE MRI of the pelvis and hips for assessment of osseous morphology in an athlete. **(A)** The coronal ZTE image demonstrates bilateral hip osseous anatomy with improved cortical definition. **(B)** Magnified view of the left hip demonstrates an osseous bump at the femoral head–neck junction in keeping with CAM morphology, an increased alpha angle of 71°, and an os acetabuli (arrow).

Hip and/or groin pain are common in athletes and represent a frequent cause of reduced performance and time lost from sport [15]. Accurate assessment of hip morphology is essential when evaluating femoroacetabular impingement syndrome. Appropriate imaging is required to assess cam morphology, pincer morphology, and features of hip dysplasia [16]. Adding a ZTE sequence to standardized pelvic or hip MRI protocols enables multiplanar and three-dimensional evaluation of hip morphology (Figure 10) and associated bony abnormalities, such as os acetabuli, with image quality comparable to CT [17].

**Figure 11:**
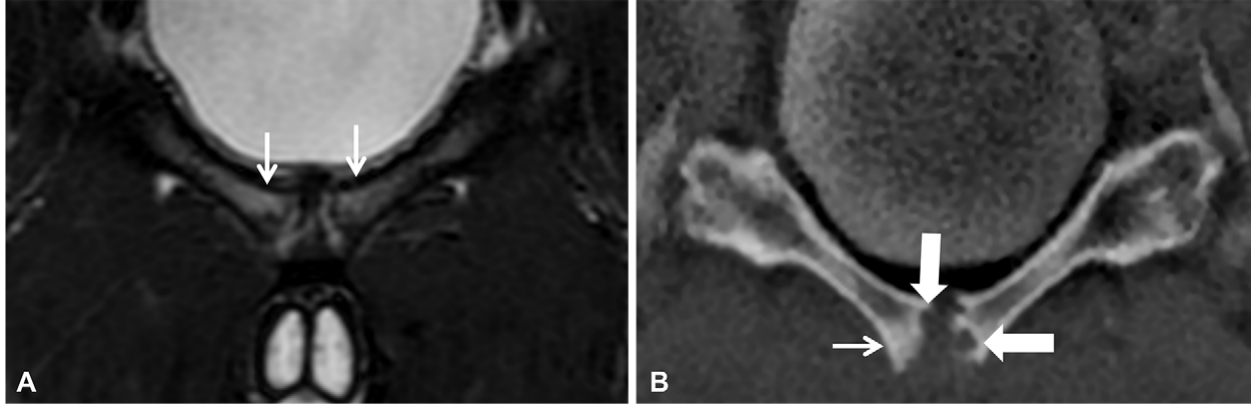
Coronal MRI of the pelvis in a 21-year-old male sub-elite football player demonstrating pubic apophysitis. **(A)** The coronal T2 fat-suppressed image demonstrates bilateral bone marrow edema at the pubic symphysis, more pronounced on the right (arrows). **(B)** The coronal ZTE image demonstrates widening of the pubic symphysis with adjacent sclerosis (arrow) and focal irregular delineation of the articular margins (bold arrow).

Pubic-related groin pain represents another important cause of symptoms in athletes and often requires evaluation of the pubic symphysis and adjacent structures. The assessment focuses on bone stress, cortical irregularity, sclerosis, and apophyseal or enthesis-related changes, with consideration of skeletal maturation in younger athletes [18]. Figure 11 illustrates apophysitis in a 21-year-old male sub-elite football player.

**Figure 12:**
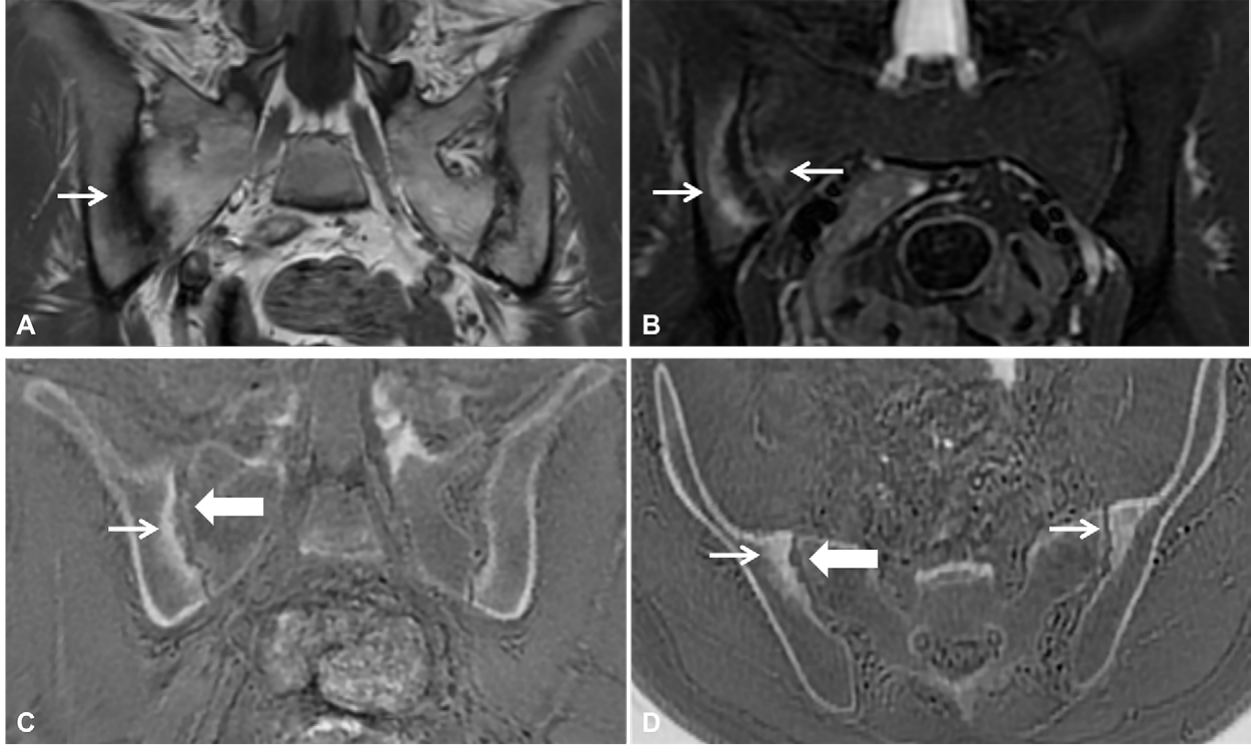
MRI of the pelvis in a 32-year-old male runner presenting with low back and buttock pain radiating to the right leg. **(A)** Oblique coronal T1-weighted image demonstrates irregular and blurred articular margins of the right sacroiliac joint with subchondral low signal intensity (arrow). **(B)** Oblique coronal T2 fat-suppressed image demonstrates extensive bone marrow edema adjacent to the right sacroiliac joint. **(C, D)** Oblique coronal and axial ZTE images show sclerosis (arrow) and erosions (bold arrow), orienting the diagnosis to inflammatory sacroiliitis, which was proven after rheumatologic investigation.

Adding ZTE to conventional MRI may also be useful for the differential diagnosis of sports injuries. ZTE has been shown to be as sensitive as CT for detecting structural abnormalities, such as erosions, sclerosis, and subtle ankylosis, in axial spondylarthritis and improves diagnostic assessment compared with conventional MRI sequences in the correct clinical context of inflammatory low back pain (Figure 12) [19, 20].

## Conclusion

ZTE MRI extends the capabilities of conventional musculoskeletal MRI by enabling CT-like visualization of cortical bone and mineralized tissue within a single examination. Its application in athletes enhances the assessment of osseous abnormalities across a wide range of sports-related conditions and supports more comprehensive, efficient diagnostic imaging.

## References

[r1] Sandberg JK, Young VA, Yuan J, Hargreaves BA, Wishah F, Vasanawala SS. Zero echo time pediatric musculoskeletal magnetic resonance imaging: Initial experience. Pediatr Radiol. 2021;51(13):2549–2560. 10.1007/s00247-021-05125-5. 34156504

[r2] Chong LR, Lee K, Sim FY. 3D MRI with CT-like bone contrast—An overview of current approaches and practical clinical implementation. Eur J Radiol. 2021;143:109915. 10.1016/j.ejrad.2021.109915. 34461599

[r3] More SS, Zhang X. Ultrashort echo time and zero echo time MRI and their applications at high magnetic fields: A literature survey. Investig Magn Reson Imaging. 2024;28(4):153–173. 10.13104/imri.2024.0009. PMC12685016 41368472

[r4] Teixeira PAG, Kessler H, Morbee L, et al. Mineralized tissue visualization with MRI: Practical insights and recommendations for optimized clinical applications. Diagn Interv Imaging. 2025;106(5):147–156. 10.1016/j.diii.2024.11.001. 39667997

[r5] Aydingoz U, Yildiz AE, Ergen FB. Zero echo time musculoskeletal MRI: Technique, optimization, applications, and pitfalls. Radiographics. 2022;42(5):1398–1414. 10.1148/rg.220029. 35904982

[r6] Vuillemin V, Guerini H, Thevenin F, et al. Bone tissue in magnetic resonance imaging: Contribution of new zero echo time sequences. Semin Musculoskelet Radiol. 2023;27(4):411–420. 10.1055/s-0043-1770771. 37748464

[r7] Florkow MC, Willemsen K, Mascarenhas VV, Oei EHG, van Stralen M, Seevinck PR. Magnetic resonance imaging versus computed tomography for three-dimensional bone imaging of musculoskeletal pathologies: A review. J Magn Reson Imaging. 2022;56(1):11–34. 10.1002/jmri.28067. 35044717 PMC9305220

[r8] Lombardi AF, Ma YJ, Jang H, et al. Synthetic CT in musculoskeletal disorders: A systematic review. Invest Radiol. 2023;58(1):43–59. 10.1097/RLI.0000000000000916. 36070535 PMC9742139

[r9] Hoenig T, Hollander K, Popp KL, et al. International Delphi consensus on bone stress injuries in athletes. Br J Sports Med. 2025;59(2):78–90. 10.1136/bjsports-2024-108616. 39638438

[r10] Kiuru MJ, Pihlajamaki HK, Ahovuo JA. Bone stress injuries. Acta Radiol. 2004;45(3):317–326. 10.1080/02841850410004724. 15239429

[r11] Baltes TPA, Dalansi F, Al-Naimi MR, et al. The prevalence, size, and anatomic location of cartilage and osteochondral lesions in athletes with an acute ligamentous ankle injury. Am J Sports Med. 2025;53(9):2173–2180. 10.1177/03635465251344187. 40503595 PMC12235055

[r12] Flanigan DC, Harris JD, Trinh TQ, Siston RA, Brophy RH. Prevalence of chondral defects in athletes’ knees: A systematic review. Med Sci Sports Exerc. 2010;42(10):1795–1801. 10.1249/MSS.0b013e3181d9eea0. 20216470

[r13] van Bergen CJ, van den Ende KI, Ten Brinke B, Eygendaal D. Osteochondritis dissecans of the capitellum in adolescents. World J Orthop. 2016;7(2):102–108. 10.5312/wjo.v7.i2.102. 26925381 PMC4757654

[r14] Lombardi AF, Guma M, Chung CB, Chang EY, Du J, Ma YJ. Ultrashort echo time magnetic resonance imaging of the osteochondral junction. NMR Biomed. 2023;36(2):e4843. 10.1002/nbm.4843. 36264245 PMC9845195

[r15] Weir A, Brukner P, Delahunt E, et al. Doha agreement meeting on terminology and definitions in groin pain in athletes. Br J Sports Med. 2015;49(12):768–74. 10.1136/bjsports-2015-094869. 26031643 PMC4484366

[r16] Mascarenhas VV, Castro MO, Rego PA, et al. The Lisbon Agreement on femoroacetabular impingement imaging—part 1: Overview. Eur Radiol. 2020;30(10):5281–5297. 10.1007/s00330-020-06822-9. 32405754

[r17] Breighner RE, Bogner EA, Lee SC, Koff MF, Potter HG. Evaluation of osseous morphology of the hip using zero echo time magnetic resonance imaging. Am J Sports Med. 2019;47(14):3460–3468. 10.1177/0363546519878170. 31633993

[r18] van Ovost A, Hanff DF, Serner A, van Klij P, Agricola R, Weir A. Radiographic assessment of the pubic symphysis in elite male adolescent football players: Development and reliability of the Maturing Adolescent Pubic Symphysis (MAPS) classification. Eur J Radiol. 2023;167:111068. 10.1016/j.ejrad.2023.111068. 37666074

[r19] Zhang Z, Wang J, Li Y, et al. Bone assessment of the sacroiliac joint in ankylosing spondylitis: Comparison between computed tomography and zero echo time MRI. Eur J Radiol. 2024;181:111743. 10.1016/j.ejrad.2024.111743. 39341167

[r20] Jans LBO, Chen M, Elewaut D, et al. MRI-based synthetic CT in the detection of structural lesions in patients with suspected sacroiliitis: Comparison with MRI. Radiology. 2021;298(2):343–349. 10.1148/radiol.2020201537. 33350891

